# *VDR* and *CYP24A1* Expression Analysis in Iranian Relapsing-Remitting Multiple Sclerosis Patients 

**DOI:** 10.22074/cellj.2017.4192

**Published:** 2017-08-19

**Authors:** Hashem Sadeghi, Mohammad Taheri, Elham Sajjadi, Abolfazl Movafagh, Shahram Arsang-Jang, Arezou Sayad

**Affiliations:** 1 Department of Medical Genetics, School of Medicine, Shahid Beheshti University of Medical Sciences, Tehran, Iran; 2 Department of Hematology, School of Paramedical Sciences, Shahid Beheshti University of Medical Sciences, Tehran, Iran; 3 Department of Medical Genetics, School of Medicine, Cancer Research Centre, Shahid Beheshti University of Medical Sciences, Tehran, Iran; 4 Department of Epidemiology and Biostatistics, Faculty of Health, Qom University of Medical Sciences, Qom, Iran

**Keywords:** *VDR*, *CYP24A1*, Expression, Multiple Sclerosis, Real Time-Polymerase Chain Reaction

## Abstract

**Objective:**

Multiple sclerosis (MS) is a common disease of the central nervous system. This disease may be initiated by either vitamin deficiency or triggered by abnormality in *CYP24A1* and vitamin D receptor.

**Materials and Methods:**

In this case-control study, the expression of genes encoding vitamin D receptor (*VDR*) and *CYP24A1* in relapsing-remitting MS (RR-MS) patients was compared with normal individuals in the Iranian population. RNA from whole blood of 50 RR-MS patients (HLA-DRB1*15-negative and responders to interferon-beta with a normal vitamin D level) and 50 normal controls was extracted. The levels of *CYP24A1* and *VDR* expression were measured using real-time quantitative polymerase chain reaction.

**Results:**

The RR-MS group had a significantly more than 2 times higher expression level of *VDR* than the normal group (P=0.04). On the other hand, there was a 0.89 times decrease in the expression level of *CYP24A1* in RR-MS patients which was not statistically significant. There was no linear correlation between the risk of expanded disability status scale of Kurtzke (EDSS) and the expression level of either *CYP24A1* or *VDR*. In addition, the expression level of *CYP24A1* or *VDR* was not correlated with the duration of the disease.

**Conclusion:**

Up-regulation of *VDR* is likely to happen in RR-MS patients in the Iranian population. We did not observe a gene expression-phenotype correlation for *CYP24A1* which may be due to limited statistical power as a result of the small sample size. Although the individuals taking part in this study had normal levels of vitamin D, the increase in *VDR* expression levels may perhaps be a response to a defect in vitamin D processing. Another possibility is that despite an increase in *VDR* expression level, factors such as micro-RNAs may result in their deactivation while an increase in *VDR* expression level can be seen as a compensatory response. Of course, further studies are required to identify the mechanism of action of vitamin D by analyzing genes involved in its signaling pathway, particularly *VDR* and *CYP24A1*.

## Introduction

Multiple sclerosis (MS) is as a complex autoimmune inflammatory disease of the central nervous system which leads to myelin injury. MS, as an autoimmune disease, may be initiated by genetic and environmental factors (1-5). Among environmental factors, research in the past decade has been focused on the association between vitamin D deficiency and the risk of MS (6). Vitamin D is derived from ultraviolet B (UVB) (7) and dietary habits (8, 9). MS is more prevalent in environments lacking sufficient UVB. This suggests that geographical location serves as a factor for vitamin D synthesis (10). The inactive form of vitamin D (25-hydroxyvitamin D) is hydroxylated to its active form (1,25-dihydroxyvitamin D) by 25(OH)D-1alfa-hydroxylase (CYP27B1) (11). The active form of vitamin D then binds to the intracellular vitamin D receptor (*VDR*) and induces the expression of 1,25(OH)2D- 24-hydroxylase (24-OHase; CYP24A1) which initiates the degradation of the physiologically active form of vitamin D3 (12).

The role of vitamin D on MS has been highlighted in numerous epidemiologic studies and related fields (13). However, the mechanisms by which vitamin D may affect MS are not known. Given that genetic factors may affect MS, we have studied genetic variation in HLA and cytokine genes, and have analyzed expression of genes encoding TNFrelated apoptosis inducing Ligand (TRAIL) and matrix metalloproteinase-9 (MMP9) in Iranian patients with MS previously (14-18). Genes encoding VDR and CYP24A1, as a key enzyme in vitamin D metabolism, are reported to be involved in MS in certain countries (19, 20). Also, a few gene expression studies on vitamin D metabolism in MS have been carried out thus far (21). Also, there have been a few studies with sufficient sample size on the nerve tissue, and studies analyzing these genes in the blood have been very limited in number (19- 20). Nonetheless, no such study has been done to examine the expression of these genes in the Iranian population so far. We therefore aimed to examine the expression levels of *VDR* and *CYP24A1* in RR-MS patients in Iran.

## Materials and Methods

In this case-control study, blood samples were taken from 50 relapsing-remitting MS (RRMS) patients (29 females and 21 males) and 50 healthy as a control group (31 females and 19 males).

Magnetic resonance imaging (MRI) was used to identify MS in patients based on the McDonald criteria (22, 23). All the patients taking part in this study were HLA-DRB1*15- negative, clinically stable and responsive to interferon-beta. Cinnovex™ was administered to all patients as part of their treatment. Vitamin D levels were shown to be normal in both groups.

### Blood sampling

For each individual, 5 ml of peripheral blood was obtained. The local Ethics Committee of Shahid Beheshti University of Medical Sciences (IR.SBMU.MSP.REC.1395.47) approved this study, including the blood collection procedure. Written consent was obtained from all individuals. The blood samples were collected at Iran’s MS Society Clinic.

### Quantitative real time-polymerase chain reaction

Total RNA was extracted using Geneall Hybrid-RTM blood RNA extraction kit (General Biotechnology, Korea), in line with the manufacturer’s instructions. The Average OD260/280 of the extracted RNA was 1.9 and had a concentration of 100 ng/microliter. Next, cDNA was synthesized using the Biosystems High-Capacity cDNA Reverse Transcription Kit (Applied Biosystems, USA). For designing the specific probes and primers, allele ID 7 (Premier Biosoft, Palo Alto, USA) was used. The sequences of all probes and primers are given in Table 1. Primers for *VDR* and *CYP24A1* were designed to span the exonexon junction. In addition, DNase was used to remove DNA contamination. Real-time quantitative polymerase chain reaction (PCR) was carried out in a Corbett Rotor Gene 6000 machine (Corbet Life Science) by using the BiosystemsTaqMan® Universal PCR Master Mix (Applied Biosystems, USA).

**Table 1:** The sequences of probes and primers


Gene name	Primer and probe sequences (5ˊ-3ˊ)	Primer and probe length	Product length	Accession number	Targeted splice variants	Average of Amplification efficiency

*HPRT1*	F:AGCCTAAGATGAGAGTTC	18	88	NM_000194.2	Single splice variant	1.98
	R: CACAGAACTAGAACATTGATA	21				
	FAM-CATCTGGAGTCCTATTGACATCGC-TAMRA	24				
*VDR*	F: TGGCTTTCACTTCAATGCTATGA	23	126	NM_000376.2	Variant 1	1.98
	R: CGTCGGTTGTCCTTGGTGAT	20		NM_001017535.1	Variant 2	
	FAM-ACTTCCGGCCTCCAGTTCGTATGGAC-TAMRA	26		NM_001017536.1	Variant 3	
				XM_011538720.2	variant X1	
				XM_006719587.3	variant X2	
*CYP24A1*	F: TATCGCGACTACCGCAAAGA	20	145	NM_000782.4	Variant 1	1.97
	R: CGGCCAAGACCTCATTGATT	20		NM_001128915.1	Variant 2	
	FAM-TCCGGACCCGCTGCCAGTCTT-TAMRA	21		XM_005260304.4	variant X1	
				XM_017027691.1	variant X2	
				XM_017027692.1	variant X3	
				XM_017027693.1	variant X4	


### Statistical methods

Independent sample t test was used to compare mean expression values. P values and 95% confidence interval (CI) were estimated for mean differences based on bootstrapping. Pearson correlation coefficient was used to examine whether the variables under study were correlated. The level of significance was set at 0.05. The analyses were implemented in SPSS 18 (Chicago, IL, USA).

## Results

Clinical details of MS patients and healthy ivdividuals are given in Table 2.

### *VDR* and *CYP24A1* expression levels and risk of relapsing-remitting multiple sclerosis

To compare the expression level of *VDR* and *CYP24A1* in RR-MS patients with normal individuals, the groups were defined as i. The total number of participants (regardless of age and sex) and ii. Age-based and sex-based subgroups (based on the age (<30, 30-40, >40 years old) and sex of participants respectively). The expression level of *VDR* in MS patients was significantly higher than normal individuals. This increase was limited to the total group. Sex- and age-based comparisons showed no statistically significant differences (Table 3).

### *CYP24A1* expression level

Compared with normal individuals, *CYP24A1* expression level in MS patients showed a decrease in all categories (i.e. the total and the two subgroups), however, none reached statistical significance (Table 4).

### *VDR* and *CYP24A1* expression levels are not correlated with expanded disability status scale or disease duration

The correlation between the expression levels of both genes and Kurtzke expanded disability status scale (EDSS) was measured among the RR-MS patients. There was no significant linear correlation between either *VDR* or *CYP24A1* with EDSS (Figes.1, 2).

Correlation of expression levels of both genes with disease duration was also analyzed in RR-MS patients. Similarly, no significant correlation was identified for either gene (Figes.3, 4).

**Table 2:** Demographic and clinical features of MS patients and healthy controls


Variable	MS patient	Control	P value

Female/Male [no. (%)]	29 (58%)/21 (42%)	31 (62%)/19 (38%)	0.683^a^
Age (mean ± SD, Y)	35.3 ± 3.2	34.8 ± 2.1	0.357^b^
Age range (Y)	20-65	19-62	
Age of onset (mean ± SD, Y)	28.36 ± 2.4	-	
Relapsing-remitting course (no. %)	100 (100%)	-	
Duration (mean ± SD, Y)	7.2 ± 3.1	-	
EDSS^c^ (mean ± SD)	2.98 ± 3.1	-	


^a^; Pearson Chi-square test, ^b^; Independent t test, ^c^; Expanded disability status scale (EDSS) of Kurtzke, and MS; Multiple sclerosis.

**Table 3:** *VDR* expression level in the RR-MS patient group, compared with the control group, based on age and sex of the participants


VDR expression	Control (n)	RR-MS patient (n)	Expression ratio	SE	95% CI	P value^a^

Total	50	50	2.11	0.617	0.341-3.607	0.04
Male	21	19	1.74	0.723	-0.15-2.344	0.6
Female	29	31	1.89	0.414	-0.299-2.53	0.06
<30						
Male	9	5	1.58	1.345	-0.201-3.825	0.85
Female	13	11	1.69	0.86	-0.123-4.405	0.43
30-40						
Male	7	6	1.69	1.15	-0.506-5.05	0.32
Female	9	9	1.78	1.7	-1.19-6.61	0.16
<40						
Male	5	8	1.82	1.403	-0.271-3.38	0.15
Female	7	11	2.02	0.82	-0.249-4.79	0.09


^a^; Independent t test, RR-MS; Relapsing-remitting multiple sclerosis patients, SE; Standard erorr, and CI; Confidence interval.

**Table 4:** *CYP24A1* expression level in the RR-MS patient group, compared with the control group, based on age and sex of the participants


*CYP24A1* expression	Control (n)	RR-MS patient (n)	Expression ratio	SE	95% CI	P value^a^

Total	50	50	0.74	0.891	-0.146 -6.953	0.06
Male	21	19	0.86	0.594	-0.106-2.758	0.5
Female	29	31	0.64	0.583	-0.84-2.868	0.07
<30						
Male	9	5	0.96	0.711	-0.23-4.575	0.75
Female	13	11	0.88	0.405	-0.815-3.986	0.51
30-40						
Male	7	6	0.85	0.98	-0.554-4.213	0.82
Female	9	9	0.69	0.89	-0.697- 2.827	0.82
>40						
Male	5	8	0.73	1.02	-0.281-3.86	0.69
Female	7	11	0.51	1.12	-0.274-2.91	0.11


^a^; Independent t test, RR-MS; Relapsing-remitting multiple sclerosis, SE; Standard erorr, and CI; Confidence interval.

**Fig.1:**
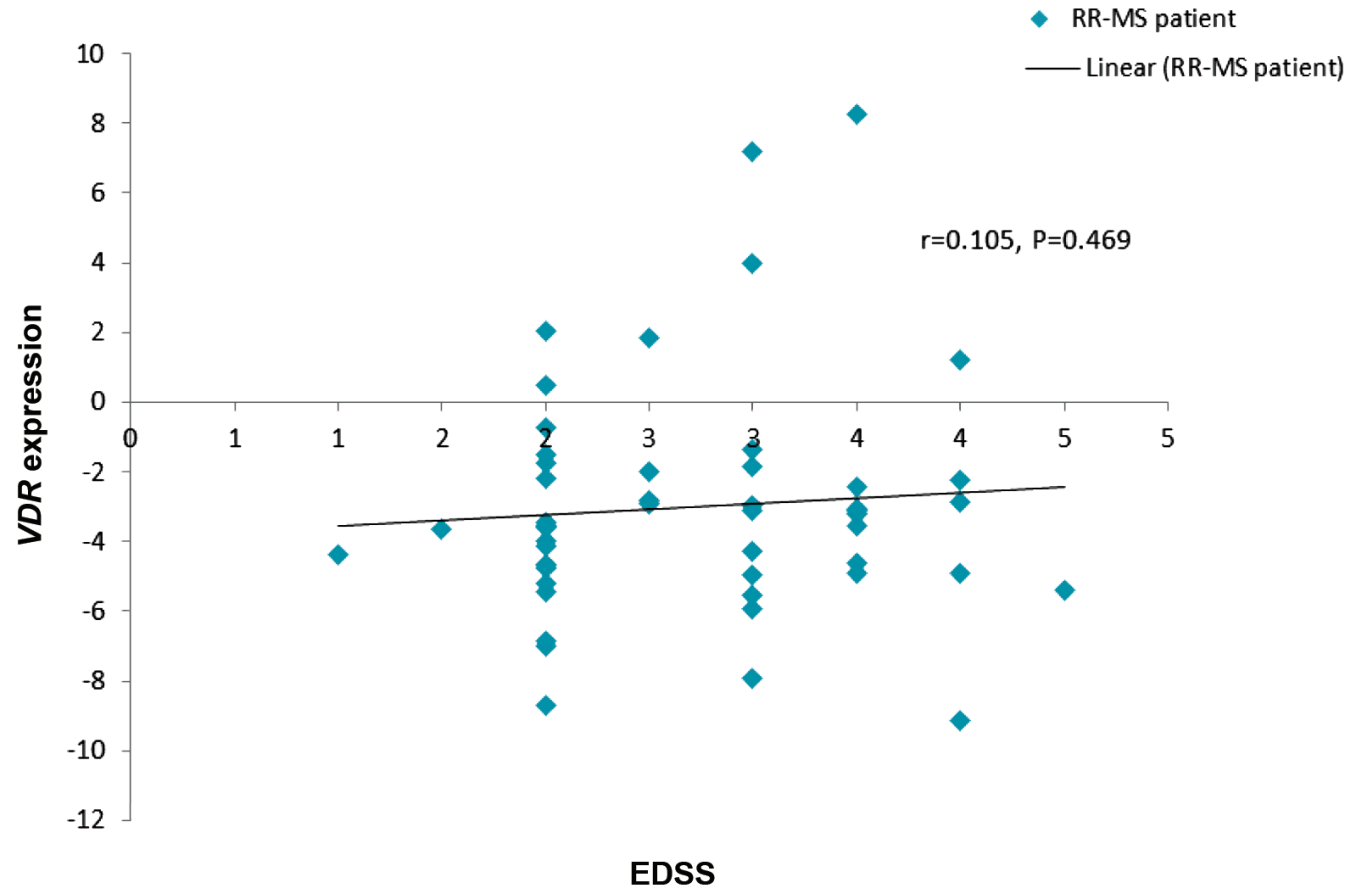
Correlation between *VDR* relative expression and EDSS in the patient group. *VDR*; Vitamin D receptor, EDSS; Expanded disability status scale of kurtzke, RR-MS; Relapsing-remitting multiple sclerosis, and r; Pearson correlation coefficient.

**Fig.2:**
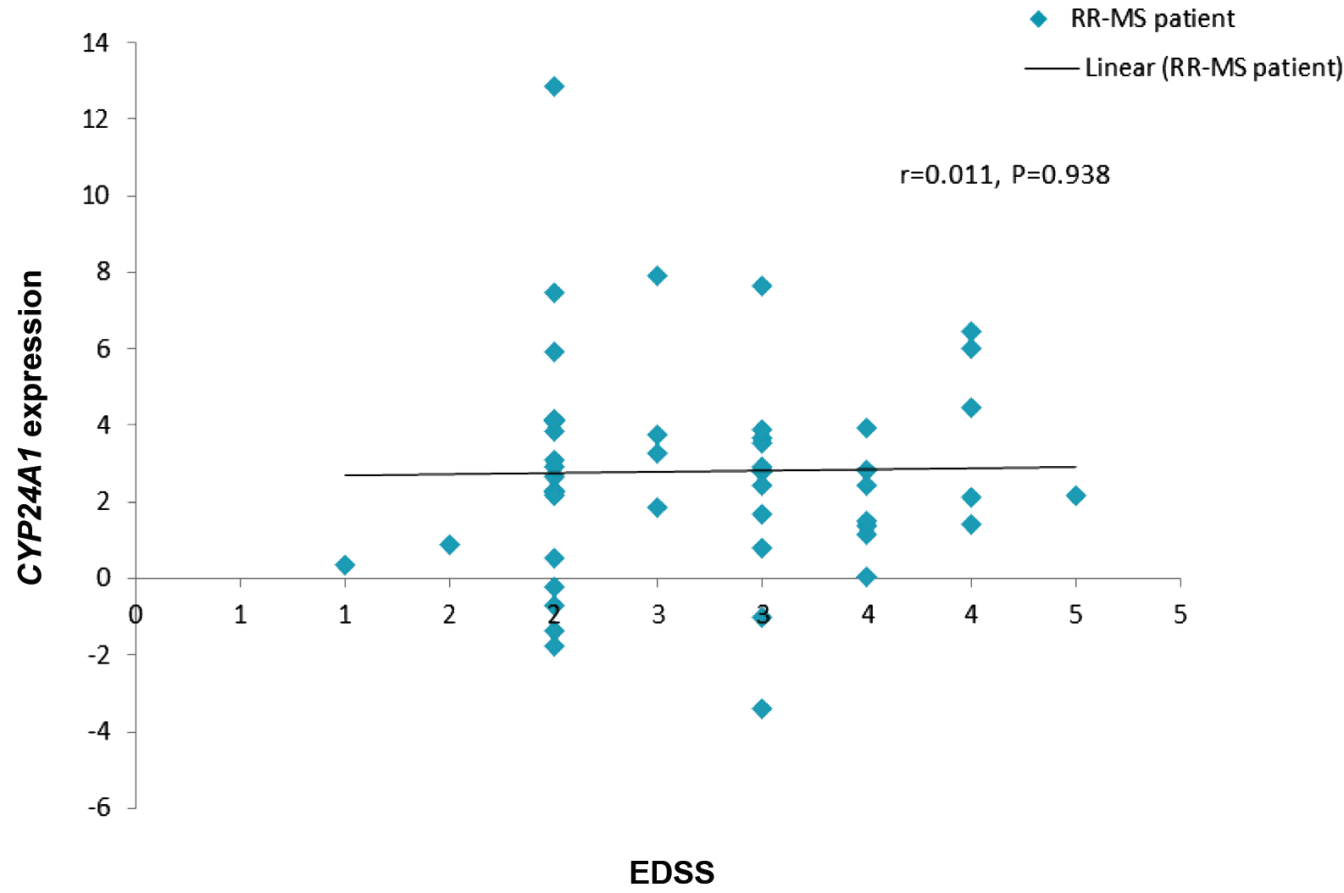
Correlation between *CYP24A1* relative expression and EDSS in the patient group. EDSS; Expanded Disability Status Scale of Kurtzke, RR-MS; Relapsing-remitting multiple sclerosis, R2; Regression EDSS, and r; Pearson correlation coefficient.

**Fig.3:**
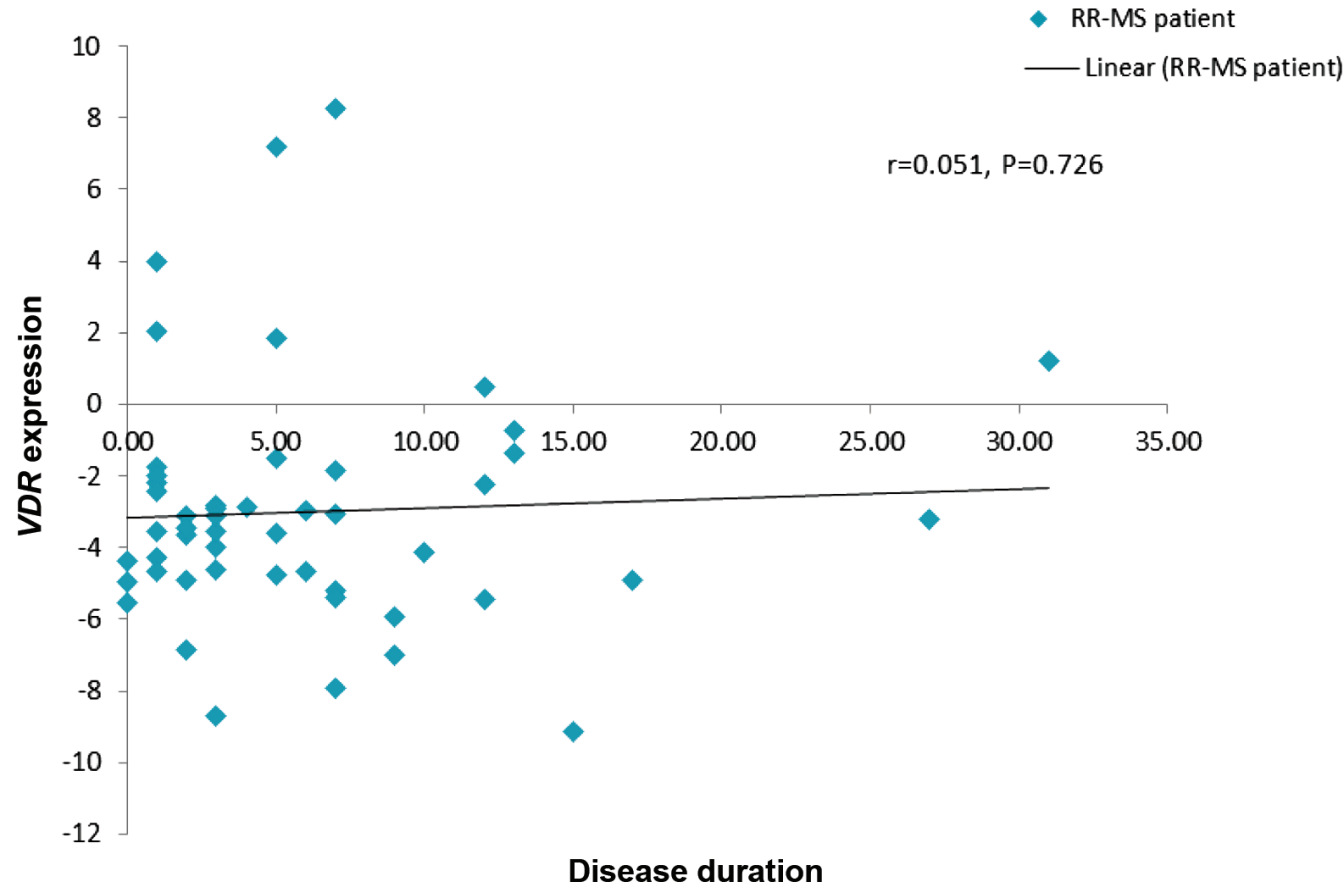
Correlation between *VDR* relative expression and disease duration in the patient group. *VDR*; Vitamin receptor, RR-MS; Relapsingremitting multiple sclerosis, and r; Pearson correlation coefficient.

**Fig.4:**
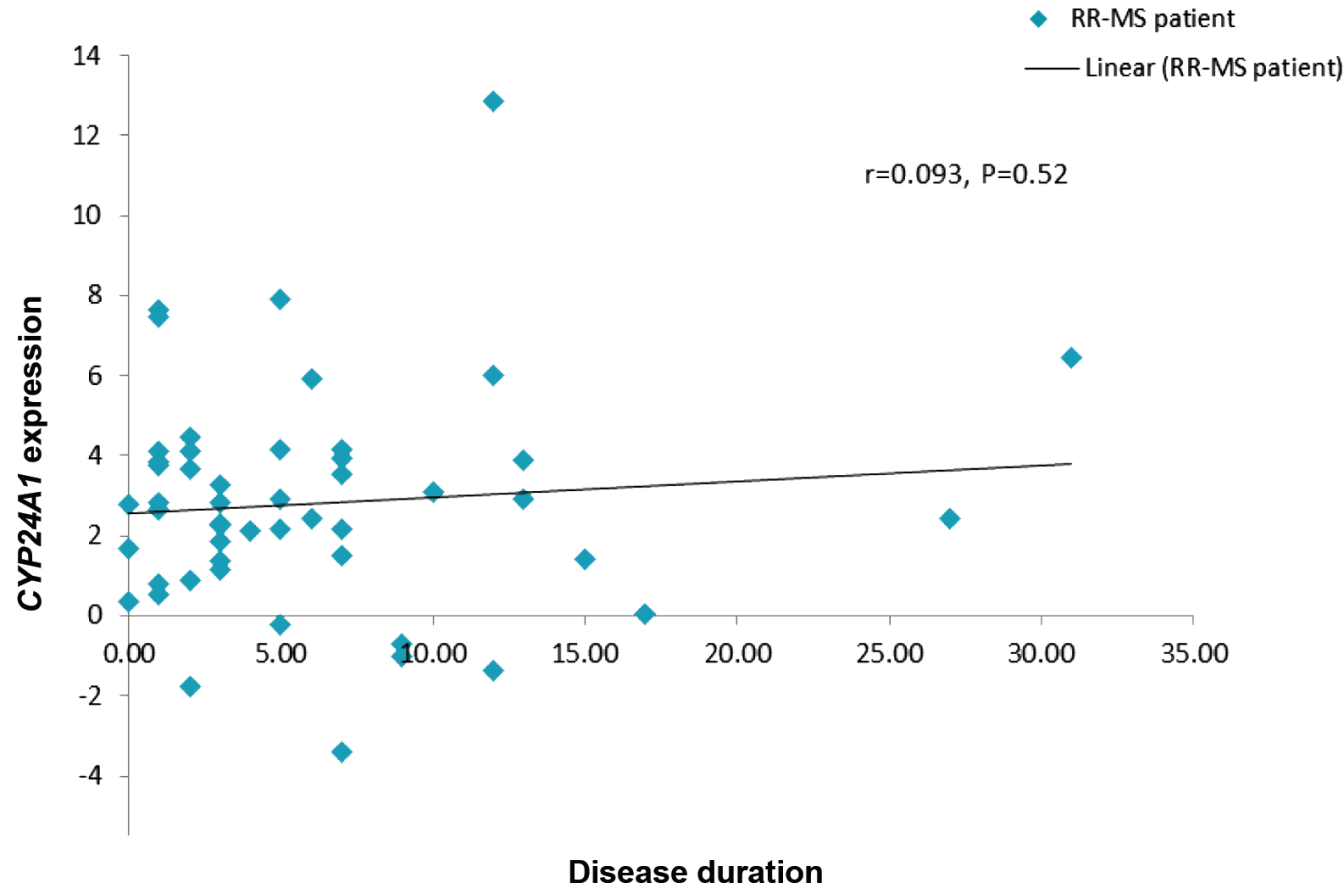
Correlation between *CYP24A1* relative expression and disease duration in the patient group. R; Pearson correlation coefficient and RR-MS; Relapsing-remitting multiple sclerosis.

## Discussion

Genetic factors have been implicated in MS onset. Besides, vitamin D deficiency may also serve as a risk factor in initiating MS. Changes in the expression of VDR and metabolizing enzymes such as CYP24A1, which is involved in the degradation of 1,25-dihydroxyvitamin D3, may thus affect MS development. To enter the cell, the active form of vitamin D binds to VDR, which is also a transcription factor. CYP24A1 in turn functions as a negative feedback and degrades the active metabolite of vitamin D. The relationship between these factors and MS has been investigated previously. For instance, Pierrot-Deseilligny and Souberbielle (24) examined the contribution of vitamin D insufficiency to the pathogenesis of MS. Ramasamy et al. (25) studied the pathogenic role of *CYP24A1* in MS, while Fukazawa et al. (19) looked at the association of vitamin D receptor gene polymorphisms with MS in the Japanese population. The current study investigated the association of *CYP24A1* and *VDR* expression levels in Iranian MS patients. Although we observed a significant increase in *VDR* expression in MS patients, the decrease in *CYP24A1* expression was not statistically significant.

It has been shown that the polymorphisms of *VDR* contribute to the risk of MS. For example, the FOKL variant in *VDR* has a different effect on the immune system with its short form, leading to a greater activation of the immune system. It can also increase *CYP24A1* expression without affecting *VDR* expression (26, 27). These polymorphisms may affect the stability and/or efficiency of the transcription of RNA, the structure of protein, the level of transcription and even their splicing (28).

Some studies have shown that the expression of this gene increases at both transcript and protein levels in active MS lesions (12). In contrast to our study, Smolders et al. (29) found no change in *VDR* expression in peripheral blood mononuclear cell (PBMC). Interestingly, they also studied CD4+ T-cell subset distribution and found a significant relationship in gene expression of their patients in IFN-γ^+^ CD4^+^ T-cell subsets. More importantly, in PBMC there was an inverse correlation between *VDR* expression and circulatory IFN-γ^+^ CD4^+^ T-cells. Based on these results, vitamin D processing by immune cells is not affected in MS; rather it has the potential to get involved in the composition of the peripheral CD4^+^ T-cell compartment. Of course, in the study by Smolders et al. (29), 35% of patients received a high level of vitamin D (high vitamin D dose, 20,000 IU/day) and their 25 (OH) D serum level was thus significantly higher than the control group. A higher serum level than in normal controls probably affects expression level of genes related to vitamin D metabolism, thereby confounding the results. This could even affect the existing correlation of gene expression levels in different PBMC cell subsets (12, 29).

The mechanism involved in the activation of the enzymatic pathway of vitamin D metabolism in PBMC or T-cells is not yet known, nor is the way such enzymes may influence the pathobiology of MS (29). Recent studies have attempted to elucidate the molecular mechanism of the immunological effects of calcidiol metabolites in patients with MS, showing that an increase in calcidiol could cause a decrease in the activity of MS. Such effects, to some extent, could be initiated by interferon-beta (IFN-β) (30). Given that vitamin D is a strong regulator of the immune system, inflammatory activities that stimulate T-helper cells toward greater inflammation may be blocked by vitamin D (31, 32). The mechanism of action of vitamin D is however complex and is likely to be influenced by various factors.

In this study, there was no significant correlation between *VDR* and *CYP24A1* expression levels and clinical findings, such as the level of physical disability in MS patients (according to the EDSS criterion) and disease duration. It is likely that the data on the amount and duration of the intake of vitamin D and serum levels of different metabolites of this vitamin may aid the interpretation of gene expression data. Also the interaction between genes and uncertainty about the transcript and protein levels increases the analytical complexity. For instance, although the people taking part in this study had normal levels of vitamin D, the increase in *VDR* expression may be a response to the defective vitamin D processing. This increase in *VDR* expression may also be due to factors such as microRNAs which may cause VDR and metabolizing enzymes down-regulation, thus the up-regulation may be seen as a compensatory response. Further studies with greater number of subjects are necessary to validate the results reported in the current work. Also, additional studies are recommended to investigate the expression level of other genes involved in vitamin D synthesis, type polymorphisms and examine protein-level expression of VDR and CYP24A1. The likelihood of such links can may also be assessed in other subsets of T cells and T-regulatory cells.

## Conclusion

The role of vitamin D in MS has been highlighted in numerous epidemiologic studies and related fields. However, the mechanism by which vitamin D affects MS is yet to be identified. All the genes that affect vitamin D metabolism belong to the genetic causes of MS. Two of the genes that play a key role in vitamin D metabolism are *VDR* and *CYP24A1*. Although *CYP24A1* was not dysregulated, up-regulation of *VDR* was observed in RR-MS patients in Iranian population. Expression analyses of the whole vitamin D pathway in RRMS patients may therefore shed further light on the underlying molecular basis of MS.

## References

[1] Peltonen L (2007). Old suspects found guilty the first genome profile of multiple sclerosis. N Engl J Med.

[2] Ebers GC, Sadovnick AD, Risch NJ (1995). A genetic basis for familial aggregation in multiple sclerosis. Nature.

[3] Ebers GC, Sadovnick AK, Dyment DD, Yee IM, Willer CJ, Risch N (2004). Parent-of-origin effect in multiple sclerosis: observations in half-siblings. Lancet.

[4] Orton SM, Herrera BM, Yee IM, Valdar W, Ramagopalan SV, Sadovnick AD (2006). Sex ratio of multiple sclerosis in Canada: a longitudinal study. Lancet Neurol.

[5] Dyment DA, Yee IM, Ebers GC, Sadovnick AD (2006). Multiple sclerosis in stepsiblings: recurrence risk and ascertainment. J Neurol Neurosurg Psychiatry.

[6] Pierrot-Deseilligny C, Souberbielle JC (2010). Is hypovitaminosis D one of the environmental risk factors for multiple sclerosis?. Brain.

[7] Calvo MS, Whiting SJ, Barton CN (2005). Vitamin D intake: a global perspective of current status. J Nutr.

[8] Holick MF (2006). High prevalence of vitamin D inadequacy and implications for health. Mayo Clin Proc.

[9] Holick MF (2007). Vitamin D deficiency. N Engl J Med.

[10] Marrie RA (2004). Environmental risk factors in multiple sclerosis aetiology. Lancet Neurol.

[11] Malloy PJ, Pike JW, Feldman D (1999). The vitamin D receptor and the syndrome of hereditary 1, 25-dihydroxyvitamin D-resistant Rickets 1. Endocr Rev.

[12] Smolders J, Schuurman KG, van Strien ME, Melief J, Hendrickx D, Hol EM (2013). Expression of vitamin D receptor and metabolizing enzymes in multiple sclerosis— affected brain tissue. J Neuropathol Exp Neurol.

[13] Sundstrom P, Salzer J (2015). Vitamin D and multiple sclerosisfrom epidemiology to prevention. Acta Neurol Scand.

[14] Sayad A, Allameh A, Sayad A, Noruzinia M, Sarzaeem A (2013). The influence of-330 IL-2 gene polymorphism on relapsing remitting and secondary progressive multiple sclerosis in Iranian patients. Neurol Asia.

[15] Sayad A, Allameh A, Sayad A, Noruzinia M, Akbari MT, Sarzaeem A (2013). The association of-475 and-631 interleukin- 2 gene polymorphism with multiple sclerosis in Iranian patients. Cell J.

[16] Sayad A (2014). The association of− 330 interleukin-2 gene polymorphism and HLA-DR15 allele in Iranian patients with multiple sclerosis. Int J Immunogenet.

[17] Taheri M, Nemati S, Movafagh A, Saberi M, Mirfakhraie R, Eftekharian MM (2016). TRAIL gene expression analysis in multiple sclerosis patients. Hum Antibodies.

[18] Yazdandoost Hamedani Sh, Taheri M, Omrani MD, Sajjadi E, Mazdeh M, Tabatabaei Panah AS, et al (2016). Up regulation of MMP9 gene expression in female patients with multiple sclerosis. Hum Antibodies.

[19] Fukazawa T, Yabe I, Kikuchi S, Sasaki H, Hamada T, Miyasaka K (1999). Association of vitamin D receptor gene polymorphism with multiple sclerosis in Japanese. J Neurol Sci.

[20] Tajouri L, Ovcaric M, Curtain R, Johnson MP, Griffiths LR, Csurhes P (2005). Variation in the vitamin D receptor gene is associated with multiple sclerosis in an Australian population. J Neurogenet.

[21] Rezaie Z, Taheri M, Kohan L, Sayad A (2016). Down-regulation of CYP27B1 gene expression in Iranian patients with relapsing-remitting multiple sclerosis. Hum Antibodies.

[22] McDonald WI, Compston A, Edan G, Goodkin D, Hartung HP, Lublin FD (2001). Recommended diagnostic criteria for multiple sclerosis: guidelines from the International Panel on the diagnosis of multiple sclerosis. Ann Neurol.

[23] Polman CH, Reingold SC, Edan G, Filippi M, Hartung HP, Kappos L (2005). Diagnostic criteria for multiple sclerosis: 2005 revisions to the “McDonald Criteria”. Ann Neurol.

[24] Pierrot-Deseilligny C, Souberbielle JC (2013). Contribution of vitamin D insufficiency to the pathogenesis of multiple sclerosis. Ther Adv Neurol Disord.

[25] Ramasamy A, Trabzuni D, Forabosco P, Smith C, Walker R, Dillman A (2014). Genetic evidence for a pathogenic role for the vitamin D3 metabolizing enzyme CYP24A1 in multiple sclerosis. Mult Scler Relat Disord.

[26] O Neill V, Asani FF, Jeffery TJ, Saccone DS, Bornman L (2013). Vitamin D receptor gene expression and function in a south african population: ethnicity, vitamin D and Fokl. PLoS One.

[27] Van Etten E, Verlinden L, Giulietti A, Ramos-Lopez E, Branisteanu DD, Ferreira GB (2007). The vitamin D receptor gene FokI polymorphism: functional impact on the immune system. Eur J Immunol.

[28] Tizaoui K, Kaabachi W, Hamzaoui A, Hamzaoui K (2015). Association between vitamin D receptor polymorphisms and multiple sclerosis: systematic review and meta-analysis of case-control studies. Cell Mol Immunol.

[29] Smolders J, Thewissen M, Theunissen R, Peelen E, Knippenberg S, Menheere P (2011). Vitamin D-related gene expression profiles in immune cells of patients with relapsing remitting multiple sclerosis. J Neuroimmunol.

[30] Munger KL, Kochert K, Simon KC, Kappos L, Polman CH, Freedman MS (2014). Molecular mechanism underlying the impact of vitamin D on disease activity of MS. Ann Clin Transl Neurol.

[31] Peelen E, Damoiseaux J, Muris AH, Knippenberg S, Smolders J, Hupperts R (2015). Increased inflammasome related gene expression profile in PBMC may facilitate T helper 17 cell induction in multiple sclerosis. Mol Immunol.

[32] Berge T, Leikfoss IS, Brorson IS, Bos SD, Page CM, Gustavsen MW (2016). The multiple sclerosis susceptibility genes TAGAP and IL2RA are regulated by vitamin D in CD4+ T cells. Genes Immun.

